# The Properties and Emulsion Stabilization of Fish Gelatin Regulated by Introducing Pectin

**DOI:** 10.3390/gels11110902

**Published:** 2025-11-10

**Authors:** Xi Zheng, Xin Feng, Yue Huang, Tao Zeng

**Affiliations:** 1 Chongqing Institute for Food and Drug Control, Chongqing 401121, China; juliezhengxi@163.com; 2 Chongqing Academy of Agricultural Sciences, Chongqing 401329, China; 3 Chongqing Sericulture Science and Technology Research Institute, Chongqing 400700, China; 4 Chongqing Food Industry Research Institute Co., Ltd., Chongqing 400042, China; zengtao19940212@163.com

**Keywords:** fish gelatin, pectin, emulsion, characterization, stability

## Abstract

In this study, the complexes (FG-P) based on fish gelatin (FG) and pectin (P) were prepared by a simple physical blending within a range of pectin concentrations (0–2%, *w*/*v*). The structure, interface, and emulsification properties of the obtained FG-P were analyzed. The binding between FG and pectin was dominated by electrostatic interaction and hydrogen bonding. Introducing pectin substantially increased the viscosity of FG-P. The water contact angle of FG-P gradually decreased with increasing pectin concentration. The highly interfacial viscosity and hydrophilicity of FG-P hindered the interfacial adsorption at the oil/water phase, thereby increasing the interfacial tension and phase angle. This was further manifested as an increase in the viscous modulus and a decrease in both the total modulus and elastic modulus. Despite the inhibition of interfacial adsorption, the unabsorbed FG-P was uniformly dispersed in the continuous phase to form a compact network structure, accompanied with improved rheological properties. Correspondingly, the emulsion precipitation phenomenon was effectively inhibited, and the stability of FG-P stabilized emulsions was improved with decreased droplet size.

## 1. Introduction

As a natural high-molecular-weight peptide polymer, gelatin consists of 50–1000 amino acids, primarily including hydroxyproline, proline, and glycine [1]. Generally, gelatin is obtained by the hydrolysis or thermal degradation of collagen derived from some mammal byproducts (e.g., skin, bone) [2]. Gelatin possesses good emulsifying, gelling, thickening, and stabilizing properties, which are broadly applied in cosmetics, food, and pharmaceutical fields [3]. Nevertheless, due to religious beliefs and the risk of diseases associated with bovine sources, there is an urgent need to develop alternatives to mammalian gelatin and expand its application [4]. Fish gelatin (FG) derived from fish skin, bones, and bellies has demonstrated promising potential in terms of religious beliefs, safety, environmental friendliness, and sustainability [5]. However, a relatively low abundance of proline and hydroxyproline content in FG limits the assembly of its triple helix structure, resulting in a relatively lower melting temperature and gel strength as well as poor emulsifying ability in comparison with mammalian gelatin [1,6].

Recently, some modification methods are expected to improve the properties of FG, including physical (e.g., ultrasound and irradiation), chemical (e.g., phosphorylation and glycosylation), biological (e.g., transglutaminase and laccase), and the combined methods [7]. Among these, introducing natural polysaccharides is deemed one of the most commonly used methods to improve the functional properties of FG [8]. Generally, natural polysaccharides can interact with protein through covalent interactions such as the Maillard reaction, and non-covalent interactions (i.e., hydrogen bonding, Van der Waals forces, electrostatic interactions, and hydrophobic interactions). Usually, these interactions effectively reduce the conformational space of the polysaccharides and decrease intermolecular distances, thus altering the properties of the resulting gel [9,10].

Pectin is widely found in plant cell walls and connected by α-galactouronic acid through an α-1,4-glycosidic bond. It is a natural anionic heteropolysaccharide with good activities such as the prevention of diabetes and obesity as well as the promotion of intestinal health [11]. Especially, pectin is extensively applied in both food and pharmaceutical fields due to its exceptional thickening, gelling, emulsifying, and stabilizing properties [12]. The complexes based on pectin and protein display good emulsification, gel properties, and stability [13]. For instance, He et al. examined the effect of pectin concentration on the gelling properties of ginkgo seed protein isolate-pectin complex gels. As the pectin concentration increased, the charge density of the complexes increased and the particle size gradually decreased, demonstrating favorable rheological properties [14]. Furthermore, citrus pectin (CP) can form electrostatic interactions with ovotransferrin fibers (OVTF). The obtained complexes can effectively stabilize emulsions, showing a smaller size, increased viscosity, and improved stability and textural performance with the inclusion of CP [15]. Likewise, the high concentration of pectin led to a soybean protein-pectin complex with a highly cross-linked structure and a stiffer oil-water interface, resulting in greater emulsion stability [16]. However, there is little information available regarding the influence of pectin on the properties of FG and the stabilization of their co-stabilized emulsions.

This study initially prepared FG-P complexes and inferred the interactions by investigating their visual appearance, viscosity, zeta potential, Fourier transform infrared spectroscopy (FTIR), wettability, interfacial tension, and interfacial dilatational rheology. Subsequently, the properties of the FG-P stabilized emulsions were investigated, including visual appearance, creaming index, optical microscope, droplet size distribution, and rheological properties.

## 2. Results and Discussion

### 2.1. Visual Appearance and Viscosity

**Figure 1:**
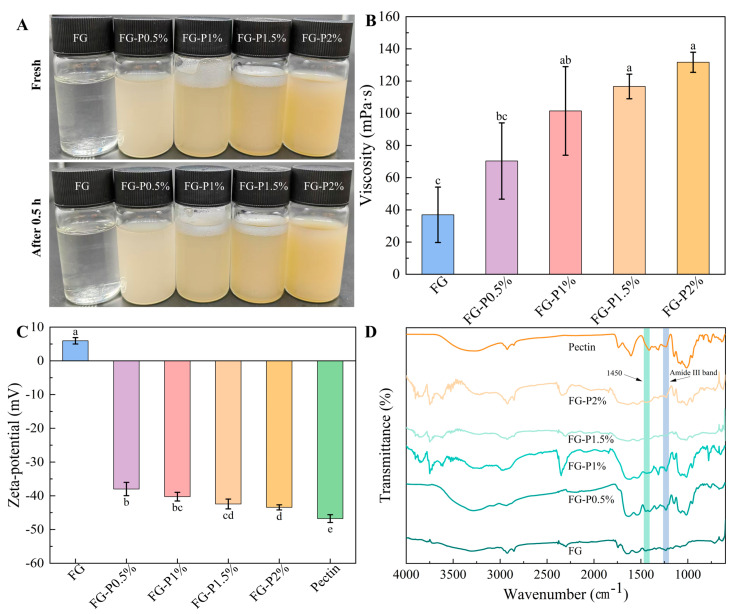
Visual appearance fresh and after 0.5 h storage at 25 °C (**A**), the changes in viscosity (**B**), zeta potential (**C**), and FTIR spectra (**D**) of FG and FG-P. Different letters (a–e) on the top columns indicate signiffcant differences (*p* < 0.05).

The visual appearance of the obtained FG-P is depicted in Figure 1A. As pectin concentration increased, the color of the FG-P solution was changed from light tan color to deep tan color, in which the apparent difference in color was attributed to the pigment inherent in citrus pectin [17]. After placing at 25 °C for 0.5 h, there was no visible phase separation or coagulation at the macroscopic level, implying a good stability of FG-P. As a commonly used food additive, pectin usually exhibits good thickening and stability properties [12]. As illustrated in Figure 1B, with the increase in pectin concentration from 0% to 2%, the viscosity of the obtained FG-P raised from 40 to 130 mPa·s, indicating that pectin can effectively address the issue of insufficient viscosity in FG. This may be due to the interaction between pectin and FG that can promote the formation of a network structure [18].

### 2.2. Zeta Potential Analysis

The zeta potential value of the obtained FG-P is depicted in Figure 1C. FG displayed a positive zeta potential value (approximately 6 mV), while pectin showed a negative zeta potential value of −47 mV. This implies that electrostatic interactions can be readily formed between FG and pectin due to their opposite charges. As the concentration of pectin rose, the zeta potential value of the FG-P progressively decreased from −38 to −47 mV. The increase in its absolute value suggested the enhanced stability of FG-P [19].

### 2.3. FTIR Analysis

FTIR spectra of the obtained FG-P are depicted in Figure 1D. The primary characteristic peaks of FG were observed at 3302 cm^−1^ (amide A band, O-H and N-H stretching vibration), 2924 cm^−1^ (amide B band, C-H stretching vibration), 1634 cm^−1^ (amide I band, C=O stretching vibration), and 1559 and 1235 cm^−1^ (amide II band and amide III band, N-H bending, C-N stretching vibration) [20]. The main characteristic peaks of pectin were located at 3258 cm^−1^ (amide A band, O-H and N-H stretching vibration), 2924 cm^−1^ (amide B band, C-H stretching vibration), and 1788 cm^−1^ (esterified carbonyl group, C=O stretching vibration) [21]. Upon the introduction of pectin, the characteristic peaks of FG shifted. The amide A band (3217–3285 cm^−1^) and amide II band (1538–1542 cm^−1^) showed a blue shift and an enhancement in hydrogen bonds. Due to the interaction between FG and pectin, the N-H/C=O groups in FG can form hydrogen bonds with the -COO-/-OH groups in pectin [22]. Furthermore, the A_III_/A_1450_ ratio is commonly utilized as an indicator to assess the integrity of the FG triple helix structure; a lower value signifies a higher degree of unwinding or a disorder in the FG structure [23]. According to Table 1, the A_III_/A_1450_ ratio gradually decreased from 0.95 to 0.64 with an increase in pectin concentration. The results suggest that the reassembly of the FG triple helix structure can be impeded by electrostatic interactions and steric hindrance, which collectively cause the unwinding of the triple helix structure and the exposure of the internal hydrophobic region [24].

### 2.4. Wettability

**Figure 2:**
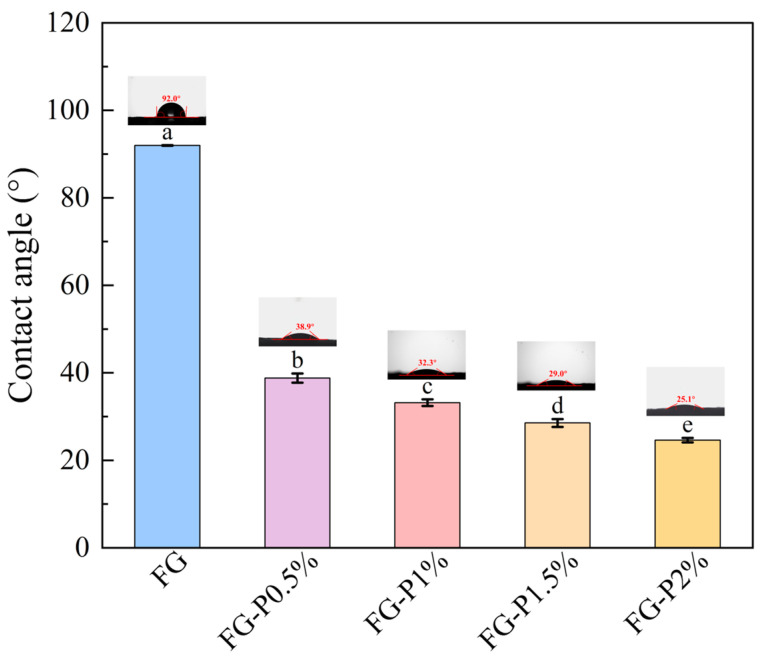
Water contact angle of FG and FG-P. Different letters (a–e) on the top columns indicate signiffcant differences (*p* < 0.05).

The three-phase contact angle is a key parameter for quantifying wettability. When the surface contact angle exceeds 90°, it exhibits hydrophobicity; when it is below 90°, it exhibits hydrophilicity [25]. As presented in Figure 2, the water contact angle of FG was 91.97°. The water contact angle of the FG-P complex decreased from 38.8° to 24.6° with increasing pectin concentration. This phenomenon indicates a transition from hydrophobicity to hydrophilicity and an enhancement of the hydrophilic characteristics. This could result from the abundant presence of polar groups such as carboxyl and hydroxyl groups in pectin, which possess good hydrophilicity [11]. The interaction between the hydrophilic groups of pectin and FG may enhance the overall hydrophilicity of the obtained FG-P [26].

### 2.5. Interfacial Tension Analysis

**Figure 3:**
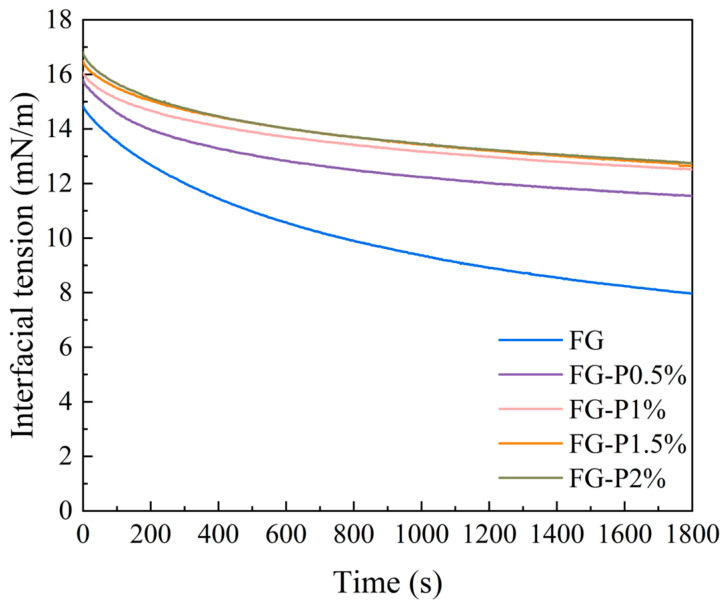
Interfacial tension of the complexes at different pectin concentrations.

The interfacial adsorption kinetics of the obtained FG-P are depicted in Figure 3. The interfacial tension of all samples decreased over time. After introducing pectin, the obtained FG-P showed an increase in interfacial tension value from approximately 8 to 13 mN/m. This indicates that the FG-P can migrate to the oil/water interface and form a dense interfacial film through interfacial adsorption and rearrangement [27]. However, the interfacial tension increased with an augmentation of pectin addition. This might be related to the wettability of FG-P. Generally, a higher wettability can promote interfacial adsorption, which enhances the interaction between particles and the aggregation of droplets, thus improving the stability of the emulsion [28]. Conversely, a higher interfacial tension might suppress interfacial adsorption. Furthermore, under high pectin concentration, the increase in apparent viscosity led to a competitive adsorption at the interface and an enhancement of the energy barrier, which inhibited the interface adsorption [29]. Even though interface adsorption was blocked, FG-P enhanced the network structure in the continuous phase. This behavior could strengthen the interaction between the interface and the continuous phase components, resulting in a three-dimensional network structure with significant spatial hindrance [30]. This enhancement was likely to increase the apparent viscosity of the emulsion and improve its stability [31].

### 2.6. Interfacial Dilatational Rheology Analysis

**Figure 4:**
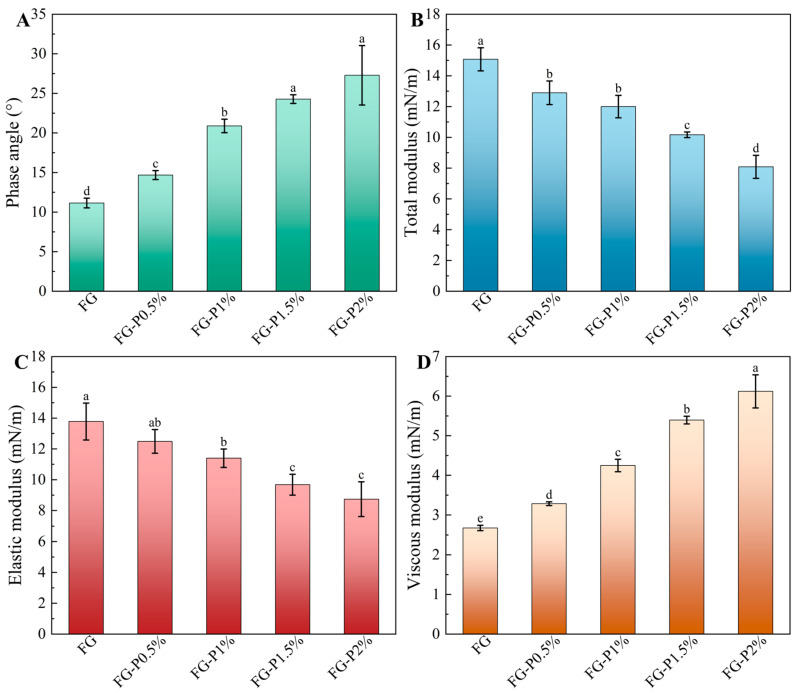
Swelling rheology of FG and FG-P: (**A**), phase angle; (**B**), total modulus; (**C**), elastic modulus; (**D**), viscous modulus. Different letters (a–e) on the top columns indicate signiffcant differences (*p* < 0.05).

Swelling rheology has been widely utilized to characterize interfacial adsorption behavior and particle interactions, and to reveal the mechanical strength and viscoelastic characteristics of interfacial adsorption layers [32]. As depicted in Figure 4, the elastic modulus of the FG-P consistently exceeded the viscous modulus, and its phase angle remained below 45°, confirming that the interfacial behavior was predominantly elastic [33]. With an increase in pectin concentration (0–2%, *w*/*v*), the phase angle increased from 11° to 27°, suggesting an increase in interfacial viscosity and a reduction in adsorption [34], which is in agreement with the results of interfacial tension adsorption. Concurrently, the total modulus and the elastic modulus decreased from 15 to 8 mN/m, 14 to 9 mN/m, and the viscous modulus increased from 3 to 6 mN/m. These changes can be attributed to when the adsorption rate of protein is decreased in the oil phase due to the formation of adsorption obstruction, thus weakening the interaction between interface particles and leading to a reduction in elastic response [29,34].

### 2.7. Visual Appearance and Creaming Index Analysis

**Figure 5:**
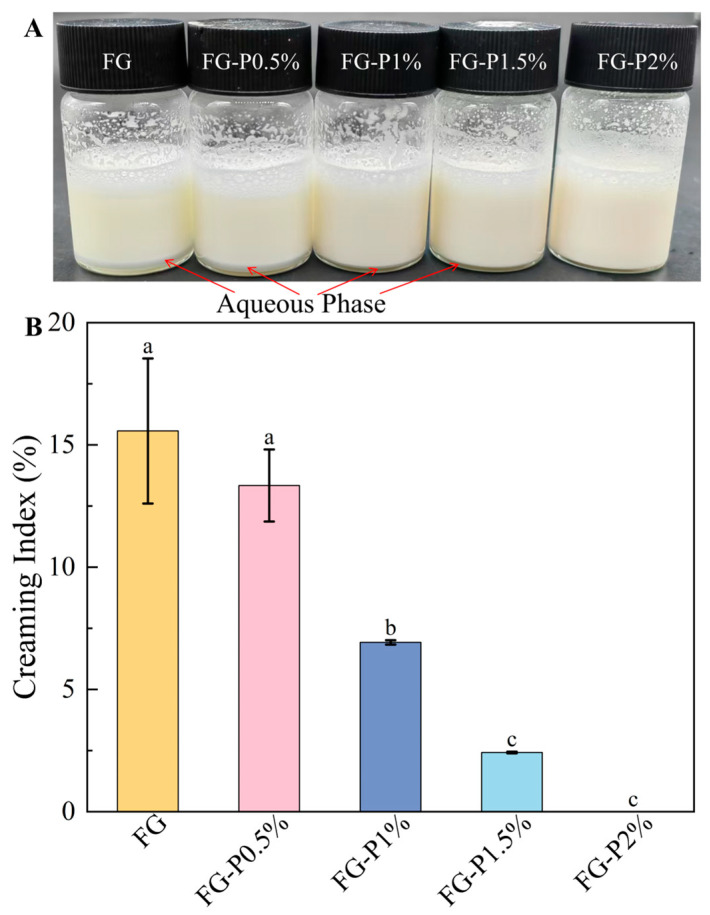
Visual appearance (**A**) and creaming index (**B**) of the fresh emulsion stabilized by the complexes at different pectin concentrations. Different letters (a–c) on the top columns indicate signiffcant differences (*p* < 0.05).

As depicted in Figure 5A, all emulsions were milky white, indicating that FG-P exhibited good emulsifying properties. As the pectin concentration increased (0–2%, *w*/*v*), the degree of water separation gradually disappeared due to the enhanced network structure, which also improves the water-holding capacity of the emulsions [29]. The creaming index test was applied to assess more accurately the stability of the emulsions (Figure 5B). The results indicated that the FG-stabilized emulsion exhibited a water separation rate of 16%, which showed an obvious phase separation due to the loose network structure and poor water retention capacity [30]. With the increase in pectin concentration (0–1.5%, *w*/*v*), the creaming index value of the emulsions progressively decreased from 13% to 2%. When the pectin concentration reached 2%, the emulsion exhibited no stratification, indicating a good emulsion stability. This improvement may be ascribed to the high concentration of pectin, which causes more FG-P to fill the continuous phase [34]. Although interfacial adsorption was somewhat inhibited, the interaction between interfacial and continuous phase components was strengthened, and the three-dimensional reticular membrane structure of the emulsion was also reinforced [26]. This can inhibit the water separation and enhance water retention capacity, consequently improving the stability of emulsions [35].

### 2.8. Optical Microscope and Droplet Size Analysis

**Figure 6:**
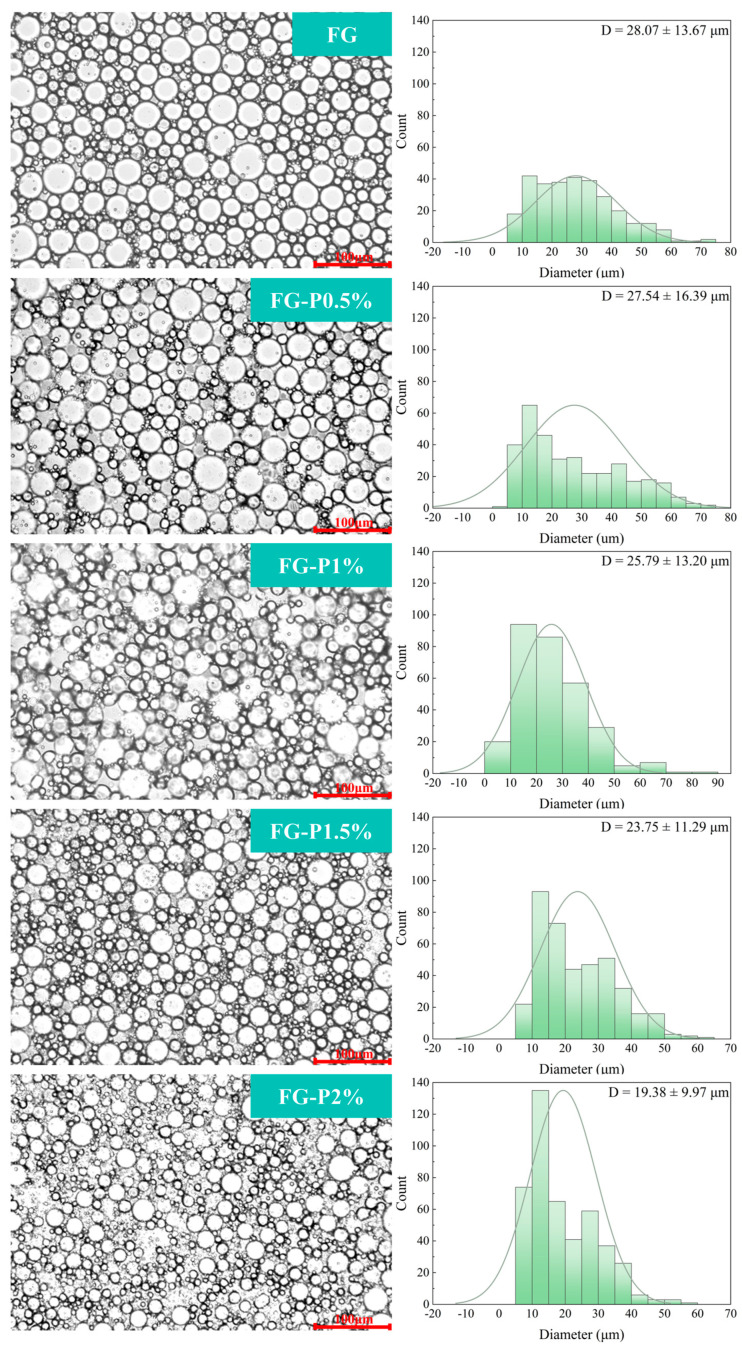
Optical microscope and size distribution of droplets of the fresh emulsion stabilized by the FG-P complexes at different pectin concentrations. The red line and number in the lower right corner of the optical microscope indicate a scale (100 μm).

Figure 6 illustrates the effect of pectin concentration on the microstructure of the FG-P stabilized emulsion. As shown in Figure 6, the large droplets of FG-stabilized emulsion were predominant. When the concentration of pectin increased, the mean droplet size progressively decreased, indicating a decrease in the large droplet population and a greater abundance of small droplets. The average diameters of the emulsions were 28 μm (FG), 27 μm (FG-P0.5%), 26 μm (FG-P1%), 24 μm (FG-P1.5%), and 19 μm (FG-P2%). The reduction in droplet size may be ascribed to more complex particles being able to participate in emulsion stabilization [21]. As the concentration of pectin increased, the surface charge density of the droplets also rose, thus enhancing electrostatic repulsion between droplets [36]. Simultaneously, the adsorption at the interface was inhibited, allowing more FG-P to fill the continuous phase. This can improve the network structure of the continuous phase and enhance the interface interaction, resulting in the formation of a robust three-dimensional network structure [26]. The combination of these factors effectively prevented droplet aggregation and enhanced the uniformity and stability of the emulsion.

### 2.9. Rheological Properties

**Figure 7:**
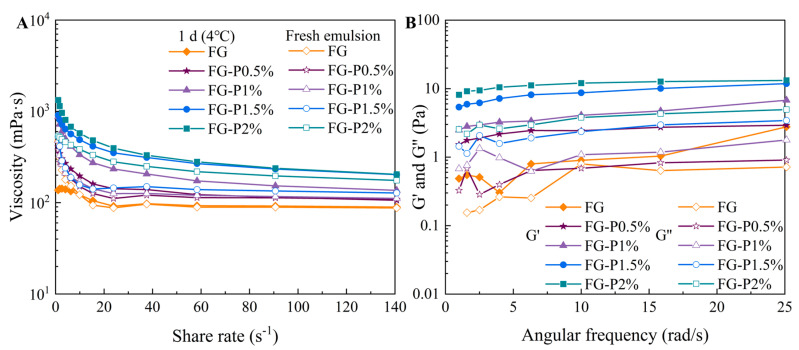
The changes by the FG-P complexes at different pectin concentrations in (**A**) apparent viscosity during shear scanning, fresh and after 1 d storage at 4 °C, and in (**B**) G′ and G″ during frequency scanning (1 d storage at 4 °C).

Figure 7A depicts the viscosity curve of the FG and FG-P stabilized emulsions. As the shear rate increased from 0 to 140 s^−1^, the viscosity of all emulsion samples decreased. For the fresh emulsion, the FG, FG-P0.5%, FG-P1%, FG-P1.5%, and FG-P2%, respectively, decreased from 470 to 90 mPa·s, 370 to 110 mPa·s, 480 to 110 mPa·s, 530 to 130 mPa·s, and 540 to 180 mPa·s. For the emulsion refrigerated at 4 °C for 1 day, the viscosity decreased from 140 to 90 mPa·s (FG), 410 to 110 mPa·s (FG-P0.5%), 870 to 140 mPa·s (FG-P1%), 910 to 200 mPa·s (FG-P1.5%), 1330 to 200 mPa·s (FG-P2%), exhibiting pseudoplastic non-Newtonian behavior. High shear forces can disrupt the network structure, causing the aggregated droplets to deform. This deformation decreased the flow resistance and viscosity as the shear rate increased [37]. With the increase in pectin addition, the viscosity of the fresh emulsion increased from 470 to 540 mPa·s, and the shear thinning phenomenon became more pronounced. The inhibition of interface adsorption allowed more particles to disperse into the continuous phase, and increased the interaction between interface particles and continuous phase particles, which formed a tight three-dimensional network structure [35]. This promoted the accumulation and mutual extrusion of oil droplets, thereby improving the viscosity of the emulsion [38]. The viscosity of the emulsions increased to 1300 mPa·s after 1 day of cold storage (4 °C), which was higher than that of the fresh emulsion. This could be because the emulsion network rearranged at low temperature to produce a denser structure, thus increasing the mechanical strength of the emulsion system [39]. As shown in Figure 7B, the G’ value of all emulsions after refrigeration storage was greater than G”, suggesting a typical elastic behavior. The values of G’ and G” showed an increase with the augmentation of pectin concentration. Correspondingly, introducing pectin resulted in the inhibition of interface adsorption and promoted the network structure formation in the continuous phase, resulting in a higher stability [40,41].

## 3. Conclusions

In this study, the regulatory role of pectin concentrations (0–2%, *w*/*v*) on the properties of FG and its stabilized emulsions was examined. Pectin can interact with FG through electrostatic attraction and hydrogen bonding and disrupt the triple helix structure of FG. The viscosity and hydrophilicity of the FG-P increased after introducing FG, which can inhibit interfacial adsorption. Consequently, the phase angle and viscosity modulus increased and both the total modulus and the elastic modulus decreased. Nevertheless, more particles were allowed to disperse into the continuous phase, which enhanced the network structure and interaction between interface particles. As pectin concentration increased, the phase separation of the FG-P stabilized emulsions was inhibited with decreased droplet size. The addition of pectin enhanced the network structure of FG-P and improved the viscosity and modulus of the FG-P emulsion. Therefore, pectin can effectively improve the viscosity, emulsification, and stability of FG.

## 4. Materials and Methods

### 4.1. Materials and Reagents

Pectin (galacturonic acid ≥ 74%, methoxyl group ≥ 6.7%, isolated from citrus) was obtained from Shanghai Aladdin Biochemical Technology Co., Ltd. (Shanghai, China). FG (from cold water fish skin) was acquired from Sigma Aldrich Corporation (St. Louis, MO, USA). Commercial soybean oil was obtained from a local market Yonghui Superstores Co., Ltd. (Chongqing, China). All other chemical reagents and solvents used were of analytical grade.

### 4.2. Preparation of FG-P

Briefly, the various concentrations (0%, 0.5%, 1%, 1.5%, and 2%, *w*/*v*) of pectin solution were first prepared by adding pectin powder into de-ionized water, followed by swelling at 90 °C for 60 min. After cooling to room temperature, FG powder was incorporated into pectin solution and dissolved at 60 °C for 10 min at a constant FG concentration (1%, *w*/*v*). The obtained FG-P were named as the FG, FG-P0.5%, FG-P1.0%, FG-P1.5%, and FG-P2.0% based on the use of pectin.

### 4.3. Visual Appearance and Viscosity of FG-P

The obtained FG-P was placed at room temperature and visually monitored every 30 min by a mobile phone (P50, Huawei, Shenzhen, China). The viscosity of FG-P was measured with a rheometer (DHR-1, TA Instruments, Surrey, UK) at 25 °C under a 20 mm plate diameter, a shear rate of 2.6 s^−1^, and a gap of 1 mm [30].

### 4.4. Zeta Potential of FG-P

The FG-P solution was diluted to 0.01% (*w*/*v*) using de-ionized water and measured with a Zeta potential analyzer (Zetasizer Nano ZS, Malvern Instrument, Malvern, UK). Subsequently, 1 mL of the diluted solution was transferred into a potential cell and tested at room temperature.

### 4.5. FTIR Measurement

The FG-P were frozen at −4 °C for 24 h prior to freeze-drying (Model FD-1-50, Beijing Boyikang Experimental Instrument, Beijing, China). The resulting lyophilized powder was subsequently placed in a Fourier transform infrared spectrometer (Spectrum100, PerkinElmer, Waltham, MA, USA) and analyzed at room temperature (25 °C) over the 4000–600 cm^−1^ range, with 4 cm^−1^ resolution and 32 scans.

### 4.6. Wettability Measurement

A water droplet was dispensed onto tablets of FG-P using a syringe. Then, the images were acquired using a video-based optical contact angle instrument (DSA100, KRUSS, Hamburg, Germany), and contact-angle data were derived through fitting analysis [28].

### 4.7. Interfacial Tension Measurement

The interfacial tension of the FG-P was determined using an interfacial tension meter (Sigma 700, Boerlin Technology, Gothenburg, Sweden). FG solution (50 mL) was transferred to a glass dish. After reaching equilibrium, an equal volume of soybean oil was introduced into the dish, and the measurement was continued until the experiment was completed. The experiment was conducted at room temperature for 30 min [42].

### 4.8. Interfacial Dilatational Rheology Measurement

The interfacial dilatational rheology of FG-P was measured using an instrument (OCA 20, Dataphysics Instruments GmbH, Filderstadt, Germany). FG-P solution (0–2%, *w*/*v*, 120 μL) was introduced into a sample cell loaded with soybean oil via a syringe, forming an ellipsoidal droplet. The droplet was subjected to periodic oscillations at room temperature (25 °C), with a 10% amplitude and a frequency of 0.1 Hz. For each oscillation cycle, five data sets, each consisting of 256 images, were acquired. The data were analyzed and computed using the OCA 20 software [33].

### 4.9. Preparation of FG-P Stabilized Emulsions

Briefly, 5 mL of FG-P solution (0–2%, *w*/*v*) was mixed with soybean oil (5 mL). After being emulsified with a homogenizer (T18, IKA Works, Inc., Wilmington, DE, USA) at a speed of 15,000 rpm for 90 s, the obtained emulsions were available for further use.

### 4.10. Appearance and Creaming Index Measurements

The fresh FG-P stabilized emulsions were placed at room temperature for 3 h and then visually inspected and photographically documented by a mobile phone (P50, Huawei, Shenzhen, China). The creaming index value was subsequently computed according to the following formula:

$$\mathrm{Creaming}\;\mathrm{index}\;(\%)=\;\frac{H_{s}}{H_{e}}\;\times \;100\%$$

where H_e_ represents the height of the entire emulsion, and H_s_ represents the height of the aqueous phase.

### 4.11. Optical Microscope and Droplet Size

The FG-P stabilized emulsions were evenly applied to a glass slide, and the microstructures of the emulsions were examined at 10× magnification at room temperature using an optical microscope (BX53, OLYMPUS, Tokyo, Japan). At least three hundred droplets in the acquired image were analyzed using ImageJ 1.53 software, and the distribution of droplet size was discretized using the Gaussian method [34].

### 4.12. Rheological Behaviors

The rheological properties of the emulsions were evaluated using a rheometer (DHR-1, TA Instruments, Surrey, UK). The apparent viscosity of both the fresh emulsion and the emulsion refrigerated at 4 °C for 1 d was measured at 25 °C under the following conditions: 20 mm plate diameter, pre-shearing time of 60 s, a shear rate swept from 1 to 140 s^−1^, and a test gap of 1 mm. Furthermore, the storage modulus (G′) and loss modulus (G″) were further determined at 25 °C with an angular frequency ranging from 1 to 25 rad s^−1^, using a 20 mm plate, a 1 mm gap, and a strain of 0.1%.

### 4.13. Statistical Analysis

All experiments were replicated a minimum of three times, and the data underwent analysis of variance (ANOVA) using SPSS Statistics 27 and Origin 2021, with results reported as mean ± standard deviation. The significance of differences between mean values was assessed using Duncan’s multiple range test (*p* < 0.05).

## Figures and Tables

**Table 1:** The peak position of the complexes at different pectin concentrations.

Sample	Amide A (cm^−1^)	Amide II (cm^−1^)	A_III_/A_1450_
FG	3302	1559	0.95
FG-P0.5%	3285	1538	0.90
FG-P1%	3217	1542	0.85
FG-P1.5%	3217	1541	0.72
FG-P2%	3219	1542	0.64

## Data Availability

The data that support the findings of this study are available from the corresponding author upon reasonable request.
